# A Tuned-RF Duty-Cycled Wake-Up Receiver with −90 dBm Sensitivity

**DOI:** 10.3390/s18010086

**Published:** 2017-12-29

**Authors:** Sadok Bdiri, Faouzi Derbel, Olfa Kanoun

**Affiliations:** 1 Department of Electrical Engineering and Information Technology, Leipzig University of Applied Sciences, Wächter Street 13, 04107 Leipzig, Germany; faouzi.derbel@htwk-leipzig.de; 2 Department of Electrical Engineering and Information Technology, Chemnitz University of Technology, Reichenhainer Street 70, 09126 Chemnitz, Germany; Olfa.Kanoun@etit.tu-chemnitz.de

**Keywords:** fast settling, wake-up receivers, event-triggered, on-demand, ultra-low power, MAC

## Abstract

A novel wake-up receiver for wireless sensor networks is introduced. It operates with a modified medium access protocol (MAC), allowing low-energy consumption and practical latency. The ultra-low-power wake-up receiver operates with enhanced duty-cycled listening. The analysis of energy models of the duty-cycle-based communication is presented. All the WuRx blocks are studied to obey the duty-cycle operation. For a mean interval time for the data exchange cycle between a transmitter and a receiver over 1.7 s and a 64-bit wake-up packet detection latency of 32 ms, the average power consumption of the wake-up receiver (WuRx) reaches down to 3 μW. It also features scalable addressing of more than 512 bit at a data rate of 128kbit/s^−1^. At a wake-up packet error rate of $10^{-2}$, the detection sensitivity reaches a minimum of −90 dBm. The combination of the MAC protocol and the WuRx eases the adoption of different kinds of wireless sensor networks. In low traffic communication, the WuRx dramatically saves more energy than that of a network that is implementing conventional duty-cycling. In this work, a prototype was realized to evaluate the intended performance.

## 1. Introduction

**Figure 1:**
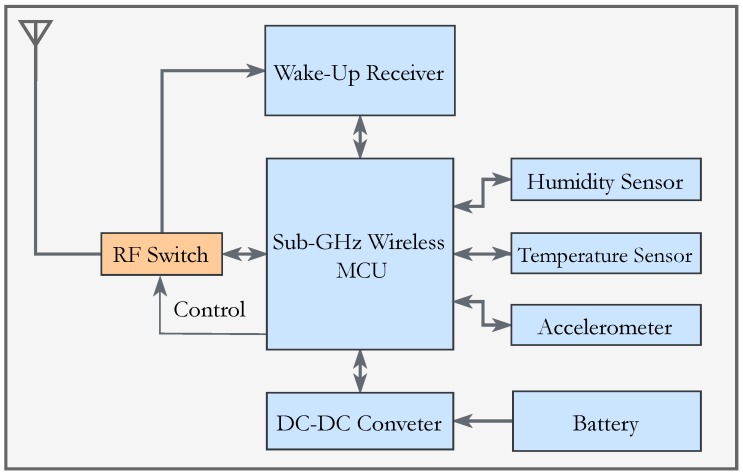
Block diagram of a typical sensor node embedded with a wake-up receiver (WuRx).

The Internet of Things has promoted the needs of wireless sensor networks (WSN) applications. Given that WSN are based on battery-powered devices, the consumed energy sets the lifetime of a WSN. The batteries are hardly replaceable in typical WSN applications, which makes controlling the consumed energy by a sensor node a critical performance parameter in the sensor node architecture. The radio receiver dominates in terms of energy usage if compared to the rest of the components. Minimizing its activity drastically saves energy and increases the entire WSN lifespan. An ultra-low power (sub-10 μW) radio receiver, referred to as the wake-up receiver (WuRx), continuously monitors the channel instead of the conventional radio. Figure 1 shows a typical configuration of a sensor node combined with the WuRx. In low traffic and less dense WSN, the usability of WuRx has more impact on energy consumption. Because of its modest architecture, the high performance in terms of sensitivity and data rate can be challenging when extreme low energy consumption is mandatory. Works like [1,2] feature ultra-low power WuRxs, but at the expense of sensitivity.

In previous literature, different architectures provide sensitivity optimizations without a major increase in energy usage. The authors in [3] introduced a WuRx, based on a passive front-end, with a digital baseband consuming 1.2 μW when monitoring a wake-up packet (WuPt). The minimum input power needed for a successful detection is −55 dBm. A more sensitive WuRx is introduced in [1,4]. The WuRx consumes 7.5 μW with a sensitivity of −60 dBm. The performance of the mentioned designs is limited by the Schottky diode noise figure [4]. Other concerned works enhance the transmission power efficiency of the WuPt. In [5], power-optimized waves are used to carry WuPt data in order to increase the radio frequency to direct current (RF-DC) conversion efficiency of the diode detector. This yields increasing rectified peak voltage while holding the incident wave’s average power constant.

In different WuRx designs, a low-noise amplifier (LNA) can be placed before the envelope detector to boost the incoming signal. This architecture is classically known as tuned-RF (TRF). It is still used in many radio systems due to its simplicity. However, increasing sensitivity will require large gain to overcome the noise figure (NF) of the following envelope detector. This requires a significant amount of power. In [6], the authors applied a duty-cycle of 0.6% on the TRF-based WuRx. It consumes an average power of 8.5 μW for a WuPt detection latency of 8.1 ms. The overall sensitivity is −73 dBm. The architecture becomes more efficient if the gain block requires less current. A more complex architecture is the superheterodyne (SH) radio. It consists of an amplifier, mixer and post amplifiers to contribute to the boosting of the signal. Additionally, an SH receiver is popular for its increased selectivity and performance compared to the TRF architecture. However, it requires an accurate local oscillator (LO) with high current demands. In [7,8], a superheterodyne front-end serves as the highly sensitive WuRx, reaching −83 dBm. To significantly reduce current usage from more than 27 mA to sub-µA, a duty-cycle of 0.1% is applied. To maintain a reasonable latency that is caused by the duty-cycling, the WuRx performs an oversampling in the nano-second scale. Nonetheless, the work does not show how all the blocks of the WuRx can sustain the very short peaks of activity. SH is probably the most complex architecture to tune in favor of the WuRx, making the room for energy savings narrower than that of other architectures [9]. The positive feedback system in super-regenerative (SR) receivers enhances a radio’s sensitivity while consuming less power than SH-based radio. The architecture relies on an RF oscillator controlled by a low frequency quench oscillator. In the linear mode of operation, the signal amplitude of the output oscillations is proportional to the amplitude of the input signal. In spite of its poor selectivity, it is employed in applications like key-less remote transmitters and AM receivers. The power demands are in the range below 1 mW, which makes it affordable for low-power applications. The shortcomings of the SR architecture are the excessive spurious emissions that do no meet the standard regulations and also the distortions it introduces to the output signal. The latter, however, is less of a concern for amplitude modulation schemes. An SR-based WuRx [10] demands 40 μW of power and emphasizes an excellent sensitivity of −97 dBm. The decoding mechanism is performed on an off-the-shelf complex programmable logic device (CPLD), requiring much higher power than most of the recently published WuRx decoders [1,3,7,8,11,12,13]. In [12], a duty-cycled SR receiver is introduced. Similar to [7,8], the reduced on-time duty of only 100 ns also reduces the inactivity time. Hence, the latency is further decreased while the average power consumption remains the same. Accordingly, the latter reaches less than 1 μW with −90 dBm sensitivity. The work, however, lacks empirical measurements when it comes to decoding efficiency or the real-world behavior against interferences. WuRx designs based on SR, SH or TR commonly consume beyond 10 μW when they are designed to be active continuously. In this work, the TR architecture is adopted while excluding SR and SH architectures because of the mentioned reasons. A duty-cycling scheme is applied by following a specific MAC protocol. The WuRx is intended to perform fast sampling, and so, the different blocks should be able to handle it.

MAC protocols for radio receivers can be divided into two categories. The first, being a synchronous MAC, requires synchronization between nodes, which defeats the purpose of embedding a WuRx in a sensor node. Therefore, only asynchronous MAC protocols are more of a concern to create a MAC-based WuRx.

**Figure 2:**
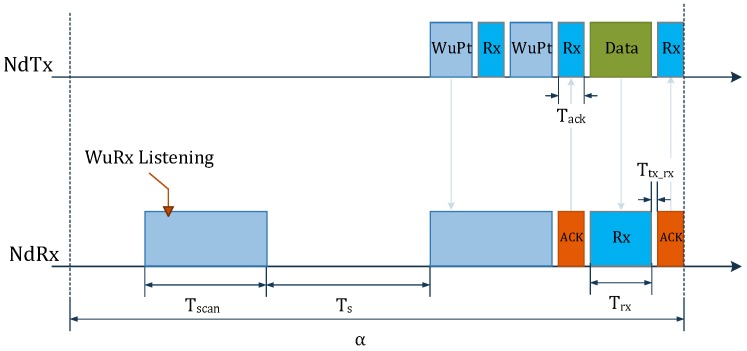
DCW-MAC scheme. WuPt, wake-up packet; Nd, node.

In most reported WuRx designs, the WuRx and the sensor node are treated separately in terms of MAC protocol execution. The WuRx listens continuously and interrupts the MCU if a WuPt is received. Afterwards, the MCU and radio operate according to a certain MAC protocol to establish a conventional link, then the WuRx switches back to a listening state. DCW-MAC, introduced in [14], is a WuRx-based MAC protocol based on X-MAC [15]. Figure 2 shows the timing diagram of a single communication cycle α between a transmitter (NdTx) and a receiving node (NdRx1), where both incorporate WuRxs. α represents the mean interval time between two transmitted data packets. At first, the WuRxs of both the transmitter and the receiver alternate between listening and sleeping states for $T_{\mathrm{SCAN}}$ and $T_{S}$, respectively. When NdTx has to initiate a data link, it starts by sending a WuPt to NdRx1, so as to wake-up the main MCU/radio. NdRx1 sends back an acknowledgment (ACK), specifying the successful reception of the WuPt. Finally, both nodes exchange data and switch to WuRx listening/sleeping mode at the end of the α cycle. It is clear that for a mean interval α, the average power consumption is governed by that of the WuRx. The latter is directly affected by the time durations $T_{\mathrm{SCAN}}$ and $T_{\mathrm{SLEEP}}$ [14]. Extensively increasing $T_{\mathrm{SLEEP}}$ reduces energy consumption, but dramatically increases the WuPt detection latency. However, by reducing $T_{\mathrm{SCAN}}$, the node can benefit from energy saving and decreased latency at the same time. Instead of listening for $T_{\mathrm{SCAN}}$ that lasts twice the WuPt’s length, the WuRx activates for as long as it allows it to identify the presence of a WuPt. This avoids unnecessary listening when there is no WuPt. Additionally, when the WuRx detects the WuPt, it remains active until the successful reception. This modification will alter the entire energy analysis of DCW-MAC. In this paper, a modified DCW-MAC (MDCW-MAC) protocol is introduced. It starts with the corresponding energy analysis. In Section 3, the WuRx’s hardware design and analysis based on simulations and interpretations are provided. Section 4 evaluates a proof-of-concept and discusses the performed tests along with the comparison to the related works. Finally, Section 5 concludes the proposed work.

## 2. MDCW-MAC

**Figure 3:**
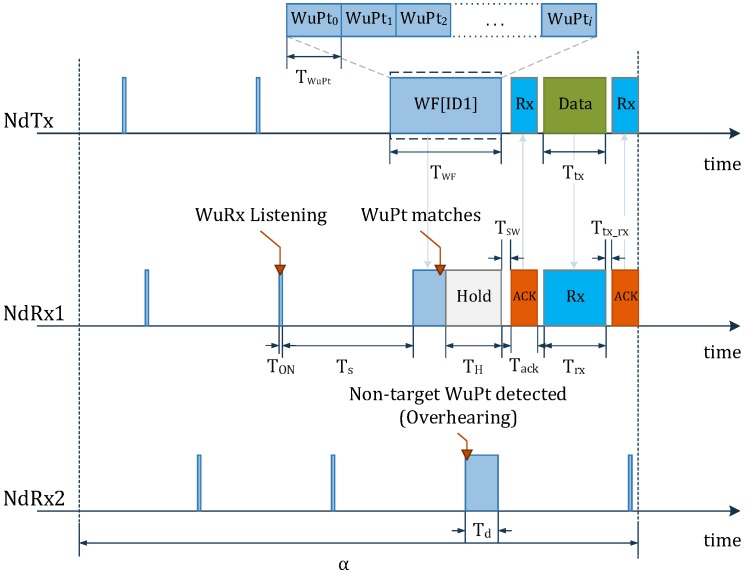
MDCW-MAC scheme.

The proposed WuRx operates intermittently by obeying an MDCW-MAC protocol (Figure 3). Consider a WSN with *N* nodes. All nodes briefly activate their own WuRxs for $T_{\mathrm{ON}}$ to check for a WuPt. When NdTx wants to initiate a communication with a NdRx1, it sends the wake-up frame (WF) as a succession of WuPts. The WuRx (WuRx1) of NdRx1 detects the WuPt, while the WuRx (WuRxn) of the non-target node (NdRxn) overhears it. The WuRx1 turns off, and the MCU and main transceiver of NdRx1 switch to the active state. The MCU waits for $T_{H}$, then sends an ACK back to the NdTx indicating that the WuPt matches with the WuRx1’s address. At the end, NdTx and NdRx1 exchange data, then terminate the communication process. NdRx1’s WuRx switches back to sleep, lasting $T_{S}$. NdRxn ignores the WuPt and continues duty-cycling its own WuRx. The entire process takes place every α. With the DCW-MAC, the transmitter switches to receiving (Rx) mode and waits for an ACK after each transmitted WuPt. This forces NdTx to stop transmitting WuPts immediately after the reception of an ACK. While packet overhead is reduced at the transmitter side, $T_{scan}$ of the WuRx cannot be further reduced if it must obey the expression in Equation (1).

The condition guarantees the reception of a WuPt.

(1) $$\begin{array}{c} T_{scan}\geq 2\;T_{\mathrm{WuPt}}+2\;T_{tx\_rx}+T_{ack} \end{array}$$

where $T_{\mathrm{WuPt}}$ is the time slot of one WuPt, $T_{tx\_rx}$ is the transition delay of the transceiver from transmission (Tx) to Rx mode and vice versa. $T_{ack}$ is the time required to receive an ACK. However, in MDCW-MAC, NdTx will only switch to Rx after sending the entire WF. The introduced WuRx incorporates a WuPt detection technique that allows $T_{\mathrm{ON}}$ to be short enough, thus reducing the latency and energy consumption. It is essential to note that for on-demand scenarios, packet communication rarely takes place. This means that the interval α is long enough when $\alpha \gg T_{\mathrm{WF}}$. By following the MDCW-MAC, the energy consumptions, $E_{\mathrm{NdTx}}$ for NdTx, $E_{\mathrm{NdTx}1}$ for NdRx1 and $E_{\mathrm{NdTxn}}$ for NdRxn in an interval α, are expressed in (2)–(4).

(2) $$\begin{array}{cc} E_{\mathrm{NdTx}} & =\alpha P_{\mathrm{SLEEPP}}+E_{l\_tx}+E_{tx} \end{array}$$

(3) $$\begin{array}{cc} E_{\mathrm{NdRx}1} & =\alpha P_{\mathrm{SLEEP}}+E_{l\_rx}+E_{rx} \end{array}$$

(4) $$\begin{array}{cc} E_{\mathrm{NdRxn}} & =\alpha P_{\mathrm{SLEEP}}+E_{l\_nrx} \end{array}$$

where $P_{\mathrm{SLEEP}}$ is the power demand of the node in the sleep state and $E_{l\_tx}$, $E_{l\_rx}$ and $E_{l\_nrx}$ are the WuRx’s average energy consumption during idle listening for the transmitter, receiver and non-target receiver, respectively. $E_{tx}$ is the energy consumed by NdTx1 during WuPt and data transmission. $E_{rx}$ represents the energy required for data reception.

During α, the WuRx’s average energy consumptions of every node $E_{l\_tx}$, $E_{l\_rx}$ and $E_{l\_nrx}$ depend on $T_{\mathrm{ON}}$ and the decoding time $T_{d}$. Assuming that the WuRx is deactivated right after finishing the decoding process, the energy models are express as follows:

(5) $$\begin{array}{cc} E_{l\_tx} & =P_{WURX}\frac{T_{\mathrm{ON}}\left(\alpha -\Delta_{tx}\right)}{T_{\mathrm{ON}}+T_{S}}, \end{array}$$

(6) $$\begin{array}{cc} E_{l\_rx} & =P_{WURX}\frac{T_{\mathrm{ON}}\left(\alpha -\Delta_{rx}\right)+T_{S}\left(T_{d}-T_{\mathrm{ON}}\right)}{T_{\mathrm{ON}}+T_{S}}, \end{array}$$

(7) $$\begin{array}{cc} E_{l\_nrx} & =P_{WURX}\frac{\alpha T_{\mathrm{ON}}+T_{S}\left(T_{d}-T_{\mathrm{ON}}\right)}{T_{\mathrm{ON}}+T_{S}}, \end{array}$$

where:

$$\begin{array}{c} \Delta_{tx}=T_{\mathrm{SW}}+T_{\mathrm{WF}}+2\;T_{ack}+3\;T_{tx\_rx}+T_{tx}\; \end{array}$$

and:

$$\begin{array}{c} \Delta_{rx}=T_{\mathrm{SW}}+T_{H}+2\;T_{ack}+2\;T_{tx\_rx}+T_{rx}\; \end{array}$$

denote the sum of activity and transition durations performed by the main transceiver and the MCU. $T_{\mathrm{SW}}$ represents the time slot required for the MCU and the radio to switch from sleep to active state. Furthermore, the energy consumptions related to data exchange or packet transmission are given by:

(8) $$\begin{array}{cc} E_{tx} & =E_{\mathrm{SW}}+P_{tx}T_{\mathrm{WF}}+2\;P_{rx}\;T_{ack}+3\;E_{tx\_rx}+P_{tx}T_{tx} \end{array}$$

(9) $$\begin{array}{cc} E_{rx} & =E_{\mathrm{SW}}+E_{H}+2\;P_{tx}T_{ack}+2\;E_{tx\_rx}+P_{rx}T_{rx} \end{array}$$

where $E_{\mathrm{SW}}$ is the energy consumption during the MCU’s transition from sleep to active. $E_{H}$ corresponds to the energy consumed during $T_{H}$. $E_{tx\_rx}$ denotes the energy needed from the transceiver to switch from Tx to Rx mode or vice versa. $P_{tx}$ and $P_{rx}$ are the power needed for packet transmission and reception, respectively. Hence, for a WSN with *N* nodes, the total energy consumption during α is expressed in Equation (10).

(10) $$\begin{array}{c} E=E_{\mathrm{NdTx}}+E_{\mathrm{NdRx}1}+\left(N-2\right)E_{\mathrm{NdRxn}} \end{array}$$

The WuRx implements the MDCW-MAC. The following section explores the WuRx’s design space.

## 3. The Wake-Up Receiver

**Figure 4:**
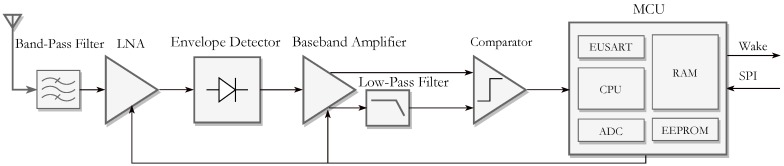
Block diagram of the proposed WuRx.

The WuRx is based on the TRF architecture. The latter requires filtering for selectivity and high RF gain to achieve high sensitivity. The bandwidth is limited comparing to other architectures (i.e., SH). The architecture is, usually, avoided for recent radio receivers. However, for specific applications like RFID, TRF fits more because of its simplicity and the inexpensive implementation [9]. The proposed WuRx incorporates a low-noise amplifier (LNA), passive square-law detector (SLD), baseband amplifiers (BBAMPS), a hysteresis comparator (HCMP) and a decoder. Figure 4 illustrates all the blocks of the WuRx. All the mentioned parts are designed to withstand the short WuPt listening period $T_{\mathrm{ON}}$. In the following sections, the design process of each peripheral is individually discussed. Let $f_{c}=868$ MHz be the carrier frequency band of both the main transceiver and the WuRx. The WuPt is modulated with on-off-keying (OOK) at baseband frequency $f_{\mathrm{BB}}$ ranging from 100 kHz to 256 kHz. Frequency-shift-keying (FSK) is the default modulation scheme for the data exchanging with a data rate $D_{\mathrm{FSK}}$.

### 3.1. The Low-Noise Amplifier

To improve the WuRx’s communication coverage, an LNA is placed after the antenna for signal amplification. Typically, RF gain demands more power when comparing to other blocks of a radio receiver chain. Fabricated with discrete parts, the LNA, designed for this WuRx, is based on [16], but consumes less power. An LNA has numerous features that set its overall performance. For typical radio receivers, it should yield the highest gain, a high stability factor and high linearity. Other criteria like the noise figure (NF), current consumption, input and output return losses should be at their minimum. Those features present several trade-offs, thus making the design process more challenging. An LNA, fabricated in a monolithic microwave integrated circuit (MMIC), provides the optimum compromise between all the mentioned figures to fit in most applications.

**Figure 5:**
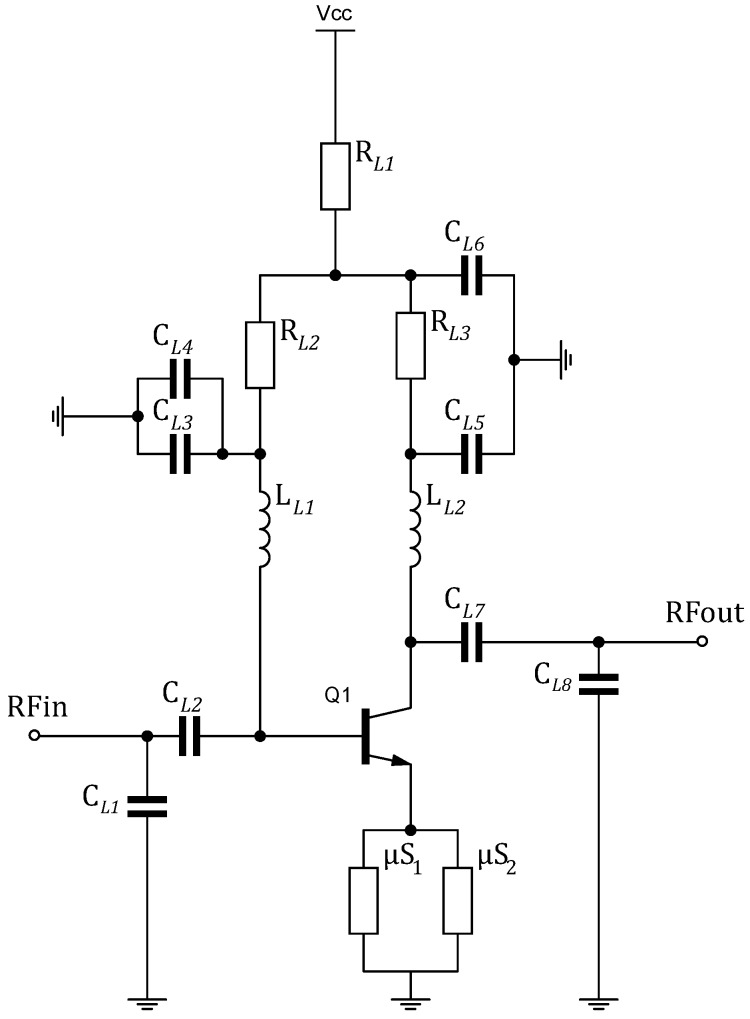
Schematic of LNA.

Commercially available MMIC-LNAs consume more than 5 mW, and even with a very low duty-cycle, they are still beyond the power requirement of the WuRx. This is directly linked to the linearity of the MMIC-LNA, as it is maximized at the cost of bias current. However, the bipolar junction transistor (BJT) creates a low cost LNA. With a minimal number of external matching and biasing networks, the BJT can quite often produce an LNA with RF performance drastically better than an MMIC. Additionally, it offers a certain degree of freedom to alternate the mentioned key parameters. This is a clear advantage for this intended WuRx design. The main concern for WuRx is enhancing the sensitivity/energy consumption tradeoff, thus low power consumption, high gain and stability LNA are prioritized among the previously mentioned characteristics. In this work, two-stage cascaded amplifiers construct the complete LNA. Although every stage should be designed differently to achieve optimal NF/linearity parameters, both stages will be identical, so as to ease the analysis and evaluation of the final LNA. A single stage is configured as a common-emitter amplifier. The LNA schematic is shown in Figure 5. $C_{L2}$ and $C_{L7}$ block DC component to be fed into the BJT. They also serve for input and output matching together with $C_{L1}$ and $C_{L8}$. $L_{L1}$ and $L_{L2}$ are RF chokes, so that they decouple the RF signal and let DC bias through. $L_{L1}$ also affects the device input impedance and the tradeoff between linearity and NF. $L_{L2}$ alters the output impedance, the gain and the general stability of the LNA. $C_{L3}$, $C_{L4}$, $C_{L5}$ and $C_{L6}$ are for RF bypassing and linearity improvement. $R_{L1}$ and $R_{L2}$ represent the resistive feedback network for biasing the LNA. $R_{L3}$ enhances the stability of the LNA at a slight cost of the gain. $\mu S_{1}$ and $\mu S_{2}$ are microstrip lines that provide inductive emitter degeneration for better linearity and easier matching. The entire cascaded LNA consumes $I_{\mathrm{LNA}}\;=\;550\;\mu A$ at $V_{\mathrm{cc}}\;=\;1.8\;V$.

Furthermore, the transducer gain $G_{tr}$ is a relevant measure of gain for a two-port system, since it takes into account the effects of both the load and source of the reflection coefficients. Providing a 2 × 2 scattering matrix for a BJT as a two-port element, $G_{tr}$, is expressed in Equation (11).

(11) $$\begin{array}{c} G_{tr}\;=\;\frac{|S_{21}{|}^{2}\left(1-\right|\Gamma_{s}{|}^{2})(1-|\Gamma_{L}{|}^{2})}{|1-\Gamma_{s}\frac{S_{11}-\Delta \Gamma_{L}}{1-S_{22}\Gamma_{L}}{|}^{2}{\left|1-S_{22}\Gamma_{L}\right|}^{2}}, \end{array}$$

where,

$$\begin{array}{c} \Delta \;=\;S_{11}S_{22}-S_{12}S_{21}\; \end{array}$$

$Z_{0}$ is the transmission line characteristic impedance. $Z_{s}$ and $Z_{L}$ are the source and load impedance seen by the input and output of the BJT device, respectively. $\Gamma_{s}=\frac{Z_{s}-Z_{0}}{Z_{s}+Z_{0}}$ and $\Gamma_{L}\;=\;\frac{Z_{L}-Z_{0}}{Z_{L}+Z_{0}}$ are the reflection coefficients associated with $Z_{s}$ and $Z_{L}$. The scattering parameters (S-parameters) are simulated using the Advanced Design System (ADS) [17] software.

**Figure 6:**
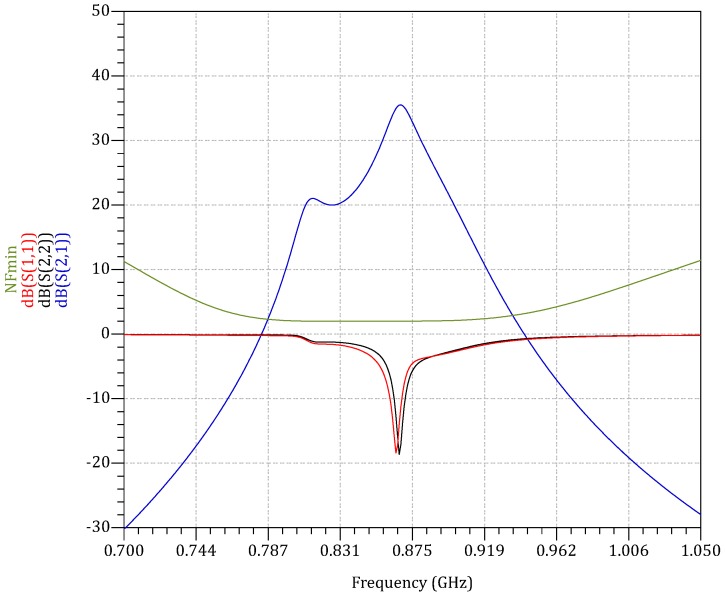
S-parameters and noise figure simulation of the two-stage LNA.

At 868 MHz, the minimum NF, ${\mathrm{NF}}_{min}=2$ dB. Additionally, the return losses $S_{11}$ and $S_{22}$ are below 10 dB for maximum power transfer. The reverse isolation $S_{12}$ is negligible. Given that $Z_{s}=Z_{L}=Z_{0}\;\approx \;50\;\Omega$, $G_{tr}\;\approx \;S_{21}$ in dB. From Figure 6, $G_{tr}=35.5$ dB. A harmonic balance simulation is performed to characterize the linearity of LNA, yielding an input-referred 1-dB compression point ${\mathrm{IP}}_{1dB}=-54\;\mathrm{dBm}$. Since non-coherent OOK is adopted for WuRx, the in-band distortions caused in the non-linear region of the LNA will not have a major impact on the detected envelope. For out-of-band signals, they are filtered at the input of the LNA by using a surface acoustic wave (SAW) filter. Therefore, high linearity is not the biggest concern, which allows for a significant reduction in bias current. Moreover, the LNA has to switch on fast enough to allow a brief WuPt listening. The LNA turn-on and turn-off time periods are mainly determined by the resistor-capacitor (RC) time constant of the biasing network.

### 3.2. The Square-Law Detector

**Figure 7:**
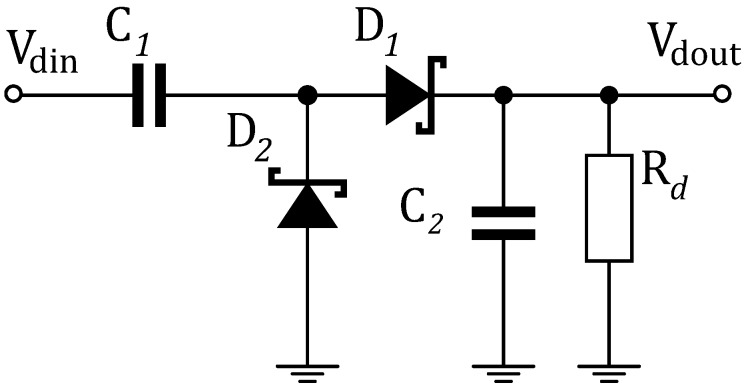
Greinacher voltage doubler as a square-law detector.

The SLD down-converts the RF signal to a baseband with much lower frequency than that of the carrier [4]. A non-linear element is the key component to perform the demodulation process. In the proposed WuRx, the SLD (Figure 7) is composed of the zero-bias Schottky diodes HSMS-2852 (Avago technologies, San Jose, CA, USA) [18].

Those provide fast switching, and they are optimized for small-signal handling of less than −20 dBm with an input signal frequency below 1.5 GHz. The diodes require no biasing, thus making the SLD fully passive. It serves to extract the WuPt from the modulated waveform. The detected signal $V_{det}$ varies proportionally with signal power $P_{din}$ at the detector’s input. The tangential signal sensitivity (TSS) is the lowest input signal power level $P_{\mathrm{TSS}}$ in watts, for which the detector will have an 8 dB signal-to-noise (SNR) ratio at the output $V_{det}$ of a single diode detector. $P_{\mathrm{TSS}}$ can be calculated as follows:

(12) $$\begin{array}{c} P_{\mathrm{TSS}}=\frac{2.5\sqrt{4kTR_{v}B_{v}}}{\gamma }, \end{array}$$

where *T* is the temperature in K, *k* is Boltzmann’s constant, $R_{v}$ is the video resistance in Ω, $B_{v}$ is the bandwidth in Hz and γ is the voltage sensitivity in V/W. Video refers to the down-converted signal (baseband), centered at 0 Hz. At 2 MHz of video bandwidth $B_{v}$, TSS = −57 dBm at room temperature. From (12), it is clear that a lower signal $B_{v}$ results in a lower detected power [19]. TSS degrades eventually with the increase of the detector’s noise floor. The root-mean-square (RMS) noise $V_{n}$ [19] generated by a single diode is given by:

(13) $$\begin{array}{c} V_{n}=\sqrt{4kTB_{v}R_{v}}, \end{array}$$

At the square-law region, the detection law obeys the relation in (14).

(14) $$\begin{array}{c} V_{det}=P_{in}\gamma \end{array}$$

A voltage detector with two diodes, where the output voltage is $V_{out}=2V_{det}$, can be represented as two resistors in series. Both represent uncorrelated noise sources. Therefore, the total RMS noise voltage becomes $\sqrt{8kTB_{v}R_{v}}$ or $\sqrt{2}V_{n}$. The detected voltages of each diode add coherently. Hence, the SNR of the two-diode envelope detector is improved by $2/\sqrt{2}=\sqrt{2}$ or 3 dB. The SLD employs the Greinacher voltage multiplier configuration. Other than the SNR improvement over a single diode detection, the input impedance $Z_{in}$ of the two RF-shunted diodes is reduced by half. Hence, the impedance matching network is easier to design. The input impedance is simulated $Z_{in}=(31.8-358.2i)\;\Omega$. An LC matching network precedes the diodes for impedance matching to the output of the LNA (50 Ω).

### 3.3. Baseband Amplifier

Placing a low noise baseband amplifier after the output of the envelope detector boosts the voltage level of the extracted envelope. The following design analysis is done on a single baseband amplifier (BBAMP).

**Figure 8:**
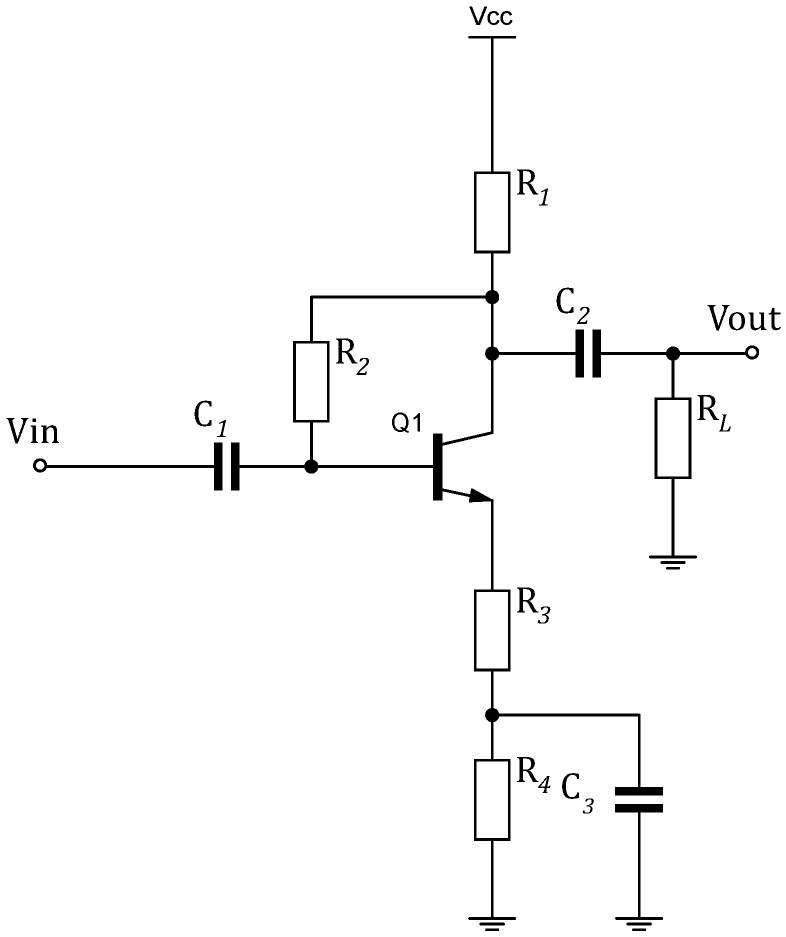
Baseband amplifier (BBAMP) configured as a common-emitter bipolar junction transistor (BJT) with collector-feedback bias.

Figure 8 shows the common-emitter (CE) configuration of the BJT-based BBAMP. In comparison with the common-collector and common-base configurations, the CE provides a very high voltage gain and medium output and input impedances. Since the gain is the main purpose of incorporating the amplifier, the CE configuration fits in the data slicer signal chain. It should be noted that the output signal of a CE amplifier has a phase shift of 180${}^{\circ }$. Biasing the transistor is a critical step for a stable amplifier. For this BBAMP, a collector-feedback biasing with emitter degeneration and a bypass capacitor are used. The base resistor $R_{2}$ is connected across the collector and the base terminals of the transistor. This means that the base voltage $V_{b}$ and the collector voltage $V_{c}$ are inter-dependent. The relation is expressed in Equation (15).

(15) $$\begin{array}{c} V_{b}=V_{c}-I_{b}R_{2}\; \end{array}$$

where

(16) $$\begin{array}{c} V_{c}=V_{cc}-\left(I_{b}+I_{c}\right)\left(R_{1}+R_{3}+R_{4}\right)\; \end{array}$$

$I_{b}$ and $I_{c}$ are the currents flowing into the base and the collector, respectively. $R_{2}$ is the resistor across voltage supply and the collector. $R_{3}$ and $R_{4}$ are series resistors connected to the emitter. Knowing that $I_{c}+I_{b}=\left(\beta +1\right)I_{b}$, from (15) and (16), $I_{c}$ can written as follows:

(17) $$\begin{array}{c} I_{c}=\frac{\beta (V_{cc}-V_{b})}{R_{2}+\left(\beta +1\right)\left(R_{1}+R_{3}+R_{4}\right)} \end{array}$$

As β varies with temperature, the quiescent point (Q-point) of the amplifier can shift beyond a desired operation point. For the collector-feedback bias configuration, $I_{c}$ can be less dependent on β in the case where $R_{2}\ll \left(\beta +1\right)\left(R_{1}+R_{3}+R_{4}\right)$. Then, the Q-point remains unchanged irrespective of the variations in the load current, causing the transistor to settle in the active region regardless of the β value. Moreover, the series $R_{3}$ + $R_{4}$ are used to enhance the amplifier’s linearity, so that larger input signals produce less distortions at the output voltage. Nevertheless, since the addition of $R_{4}$ + $R_{3}$ reduces the voltage gain $G_{\mathrm{BBAMP}}$, a capacitor $C_{3}$ is added across $R_{4}$ to form a high-pass filter (HPF). Therefore, at high frequencies, the gain $R_{3}$ is used to control $G_{\mathrm{BBAMP}}$. The expression of $G_{\mathrm{BBAMP}}$ is given by:

(18) $$\begin{array}{c} G_{\mathrm{BBAMP}}=-\frac{R_{1}R_{L}}{R_{3}\left(R_{1}+R_{L}\right)} \end{array}$$

The capacitors $C_{1}$ and $C_{2}$ block DC components and work as HPFs. In this WuRx design, the amplifier chain is composed of two-stage cascaded BBAMPS. A single BBAMP is biased with $I_{c}=7.3\;\mu A$; thus, the current consumption of the entire amplifier is $I_{\mathrm{BBAMPS}}=14.6\;\mu A$ at $V_{cc}=1.8\;V$. Furthermore, an AC simulation is performed on the amplifier to simulate its frequency response.

**Figure 9:**
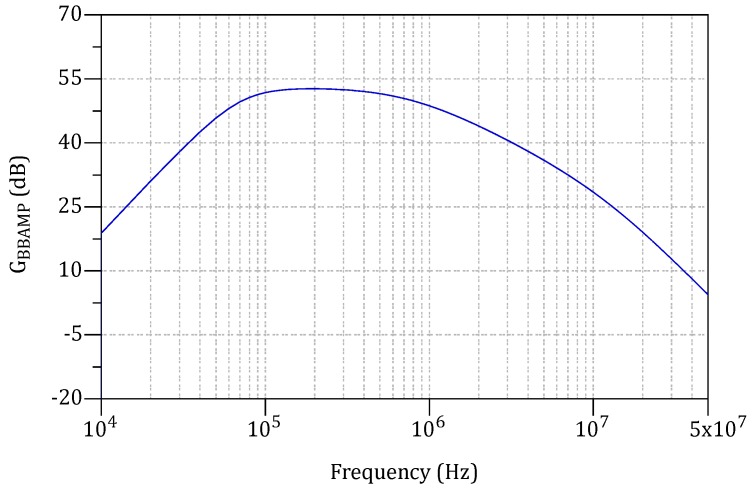
BBAMPS voltage gain vs. frequency.

Figure 9 illustrates the total voltage gain of the BBAMPS. For the frequency range 80 kHz to 770 kHz, $G_{\mathrm{BBAMPS}}\;>\;50$ dB.

### 3.4. Hysteresis Comparator

**Figure 10:**
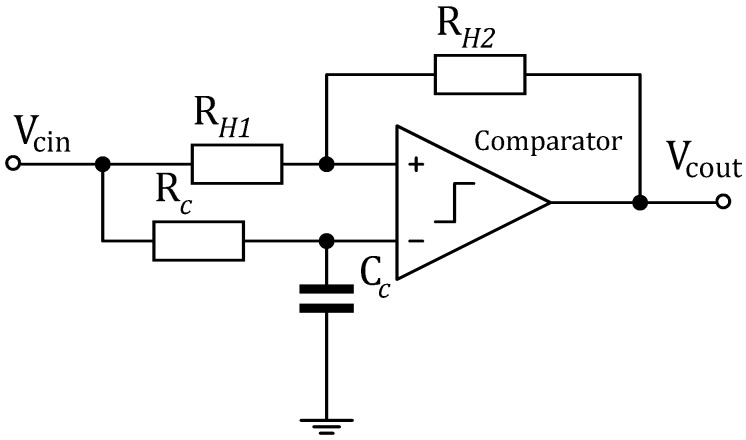
Analog to digital converter with adaptive threshold.

An analog to digital converter (Figure 10), based on a non-inverting comparator, converts the amplified signal $V_{\mathrm{AOUT}}$ to a high/low digital sequence where high represents any signal with an amplitude of more than $0.7\;V_{cc}$ and low any signal below $0.3\;V_{cc}$. An adaptive threshold $V_{ref}$, extracted from $V_{\mathrm{AOUT}}$, allows the comparator to track $V_{\mathrm{AOUT}}$ in the presence of in-band interferences.

An external hysteresis by means of a two-resistor network improves the noise immunity of the comparator. The hysteresis voltage $V_{\mathrm{Hyst}}$ creates a threshold voltage window, $V_{\mathrm{TH}+}$ and $V_{\mathrm{TH}-}$. For the comparator output $V_{cout}$ to go from low to high, the voltage input $V_{cin}$ should reach $V_{\mathrm{TH}+}$. When $V_{cin}=V_{\mathrm{TH}-}$, $V_{cout}$ goes low. Therefore, any voltage swinging that occurs within those thresholds does not affect the comparator output. $V_{\mathrm{Hyst}}$ is the difference between these transition points and can be expressed as follows:

(19) $$\begin{array}{c} V_{\mathrm{Hyst}}=V_{\mathrm{TH}+}-V_{\mathrm{TH}-}\; \end{array}$$

where:

(20) $$\begin{array}{cc} V_{\mathrm{TH}+} & =\frac{R_{H1}V_{ref}}{R_{H2}}+V_{ref} \end{array}$$

(21) $$\begin{array}{cc} V_{\mathrm{TH}-} & =\frac{V_{ref}\left(R_{H1}+R_{H2}\right)-V_{cc}R_{H1}}{R_{H2}} \end{array}$$

$V_{\mathrm{Hyst}}=50\;mV$ is chosen for this WuRx design. TLV3201 [20] (Texas Instruments, Dallas, TX, USA) is chosen to realize the threshold detector. It features an ultra-low power of $I_{\mathrm{HCMP}}=40\;\mu A$ at $V_{cc}=1.8\;V$. Given that the HCMP must cope with the $V_{cin}$ frequency, i.e., $f_{\mathrm{BB}}>100\;k\mathrm{Hz}$, the propagation delay of the comparator $t_{pd}$ must be low enough. Concerning the TLV3201, $t_{pd}=40\;ns$. The digital sequence is fed to the decoder for the WuPt correlation process.

### 3.5. Digital Baseband

An additional MCU implements the MDCW-MAC along with the decoding functionality to constitute the digital baseband (DBB) of the proposed WuRx. While it is possible to assign those tasks to the main MCU, delegating them to a second one decouples the main MCU from dealing with WuRx. It also helps the evaluation of the WuRx independently from the rest of the peripherals. The PIC12 (Microship, Chandler, AZ, USA) [21] is chosen because of its electrical characteristics, internal peripherals and the real-estate it occupies.

**Figure 11:**
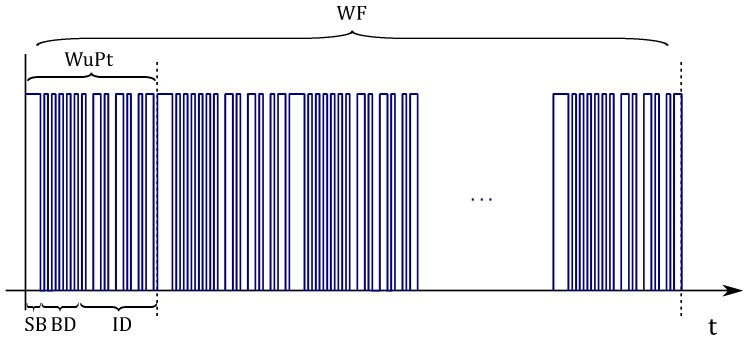
Wake-up frame structure. WF, wake-up frame.

The PIC12 wakes-up periodically for $T_{\mathrm{ON}}$ and checks if any WuPt is available. As previously mentioned, a WF is an $N_{\mathrm{WuPt}}$ repeated succession of WuPts, as shown in Figure 11, where $N_{\mathrm{WuPt}}$ can be calculated with the following expression.

(22) $$\begin{array}{c} N_{\mathrm{WuPt}}=\frac{T_{\mathrm{ON}}+T_{S}+T_{\mathrm{WuPt}}}{T_{\mathrm{WuPt}}} \end{array}$$

The WuPt bit sequence contains separation bits (SB), a baud-rate detection sequence (BD) and the WuRx address (ID). The SB sequence, $\{s_{0}\ldots s_{j-1}$, j∈N}, is composed of *j* bit. It indicates the start of WuPt and helps the decoder to localize the ID. $t_{\mathrm{SB}}$ denotes the SB sequence length. The PIC12 requires knowing $f_{\mathrm{BB}}$, so that it can properly decode the ID. The $f_{\mathrm{BB}}$ can be agreed between the decoder and the wake-up transmitter WuTx. However, some inaccuracies in the data slicer may cause $f_{\mathrm{BB}}$ to drift, thus causing bit/packet errors.

**Figure 12:**
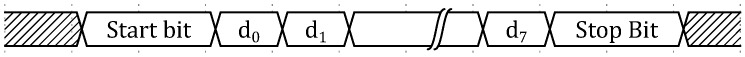
Eight-bit ID sequence diagram.

As a remedy to such an issue, the MCU can dynamically detect the $f_{\mathrm{BB}}$ within every WuPt. After detecting the SB, the PIC12 holds, waiting for the BD. The latter contains an 8-bit long character, 0x55. The consecutive rising and falling edges of such a sequence assist the PIC12 to determine $f_{\mathrm{BB}}$. The ID, as shown in Figure 12, consists of a 10k-bit sequence where $\left\{d_{0}\ldots d_{7}\right\}$ are the 8-bit pattern and 2 bit for the start and stop bits. k=2 and k=4 represent 16-bit and 32-bit IDs, respectively.

The maximum ID length $l_{\mathrm{ID}}$ depends on the capacity of the random access memory (RAM) of the decoder, excluding the amount of memory occupied by the decoder’s firmware during runtime. For instance, the PIC12 can decode more than 512 bit as it contains 256 bytes of available RAM. At last, the start and the stop bits are required to localize the pattern.

**Figure 13:**
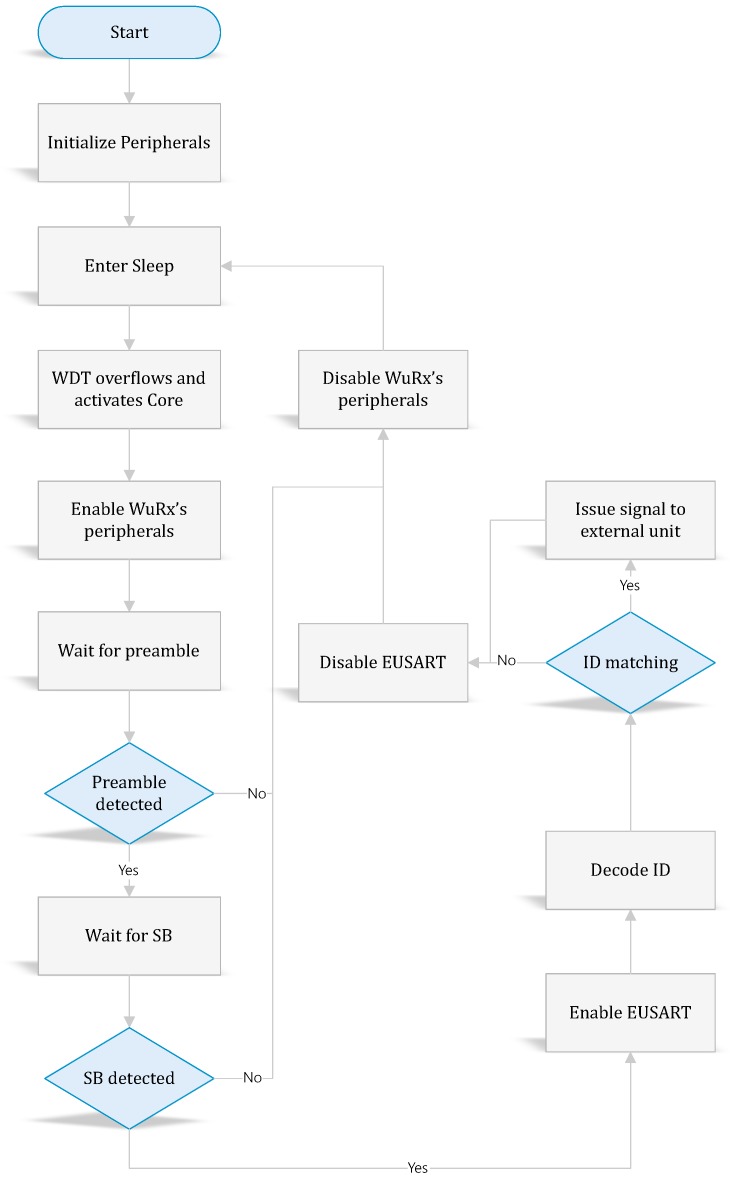
Decoder processing flowchart. SB, separation bit.

The decoder goes through different processes as illustrated in Figure 13. When the PIC12 enters the sleep state, all of its internal peripherals are automatically disabled except for the watchdog timer (WDT). By enabling the latter, the MCU can toggle between active/sleep state without the need for an external timer. The more interesting characteristic of the WDT lies in its energy consumption with only 260 nA at 1.8 V. When WDT overflows, the MCU is interrupted and switches to active state. The WDT’s time-out represents also the sleep period $T_{S}$ of the WuRx. This can be configured between 1 ms and 256 s [21].

When the MCU enables all active elements of the WuRx, it holds waiting for a WuPt till an elapsed duration of $T_{\mathrm{ON}}$. It can be seen that $T_{\mathrm{ON}}\ll T_{\mathrm{WuPt}}$. The WuPt detection process is split into two tasks. At first, the decoder has to detect a rising and a falling edge as a single pulse (i.e., ’1’ bit), so as to confirm presence of WuPt. If this is the case, it keeps all WuRx peripherals powered on and starts counting the number of rising and falling edges of the WuPt. In every count iteration, the decoder polls an input pin and waits for a certain period of time $t_{p}$, during which the maximum pulse width (i.e., $t_{\mathrm{SB}}$) should be detected. In the case of the polled amplitude-alternating signal with a frequency higher than $f_{\mathrm{BB}}$, the decoder rejects it. The above creates a certain time window for WuPt’s preamble detection. Ideally, $t_{p}$ should be slightly larger than $t_{\mathrm{SB}}$. However, to compensate for the possible variations of $f_{\mathrm{BB}}$, the following expression allows more freedom for pulse detection.

(23) $$\begin{array}{c} t_{\mathrm{SB}}<t_{p}<\frac{1}{2f_{\mathrm{BB}}}+t_{\mathrm{SB}} \end{array}$$

Therefore, $T_{\mathrm{ON}}$ depends on $t_{p}$ and the power-on time $t_{\mathrm{POWER}}$ of WuRx’s peripherals. The minimum $T_{\mathrm{ON}}$ is given in Equation (24).

(24) $$\begin{array}{c} t_{\mathrm{POWER}}+t_{p}\leq T_{\mathrm{ON}} \end{array}$$

If the counting does not reach a user-defined number $i_{c}$, the detection is considered erroneous, then the PIC12 turns-off all external peripherals and switches to sleep. Otherwise, it starts looking for SB bits, and if successfully done, it confirms the presence of a WuPt. The next process is data rate calibration. The PIC12 enables the enhanced universal synchronous asynchronous receiver transmitter (EUSART). The latter is one of the integrated peripherals and is dedicated to serial communication. After receiving the BD bits, the EUSART automatically calibrates its own clock with correspondence to $f_{\mathrm{BB}}$. Afterwards, the correlation process starts upon reception of the first ’0’ bit (start bit) after BD. The EUSART stores the $\left\{d_{0}\ldots d_{7}\right\}$ in a byte register to be read later on. The process is repeated *k* times until the processing of the entire pattern takes place. The PIC12, then, compares the pattern to the stored value. The comparison brings the decision to either issue an interrupt or not to the main MCU. In the end, the PIC12 disables the EUSART and all WuRx’s peripherals. The usage of EUSART excludes the need for a software implementation of the serial data reception.

## 4. System Evaluation

In this section, to evaluate the proposed WuRx design, all the blocks are assembled together and embedded into a sensor node.

**Figure 14:**
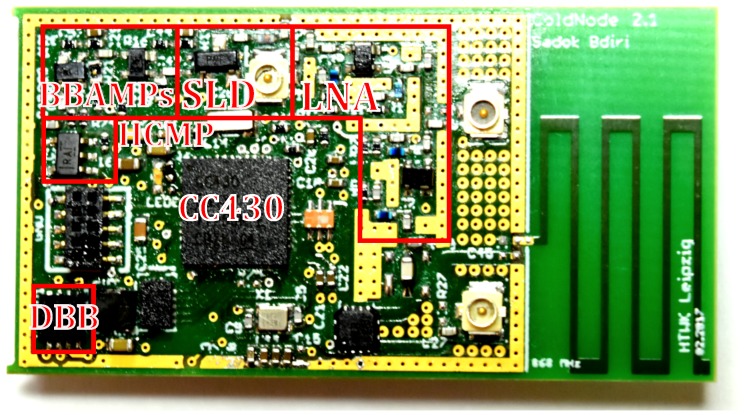
A sensor node prototype embedded with the WuRx (46.3 × 24.5 mm). SLD, square-law detector.

WuPt transmission and conventional communication are delegated to the MDCW-MAC protocol. The sensor node incorporates the wireless MCU CC430F5137 (Texas Instruments, Dallas, TX, USA) [22] (CC430), set to operate in the 868 MHz band. A single sensor node, built on a 1.55 mm four-layer printed circuit board (PCB), is shown in Figure 14. A coin cell battery with voltage $V_{bat}=3\;V$ is the main power source for the sensor node. The antenna is shared between the WuRx and the main transceiver by using the RF switch ADG918 (Analog Devices, Norwood, MA, USA) [23]. It consumes only $P_{\mathrm{RFSW}}=$ 200 nW. Additionally, a DC-DC converter can act as a buck converter to step-down the voltage to $V_{cc}$ with an efficiency of more than 90% when needed. It consumes $P_{buck}=1\;\mu W$. The buck converter’s output voltage $V_{bout}$ can be controlled externally. $P_{\mathrm{WSleep}}$ is the minimum sleep power of the WuRx. When the CC430 enters Low-power Mode 3 (LPM3) during sleep, it consumes $P_{\mathrm{MSleep}}=1\;\mu W$. Table 1 lists all power parameters of the sensor node.

**Figure 15:**
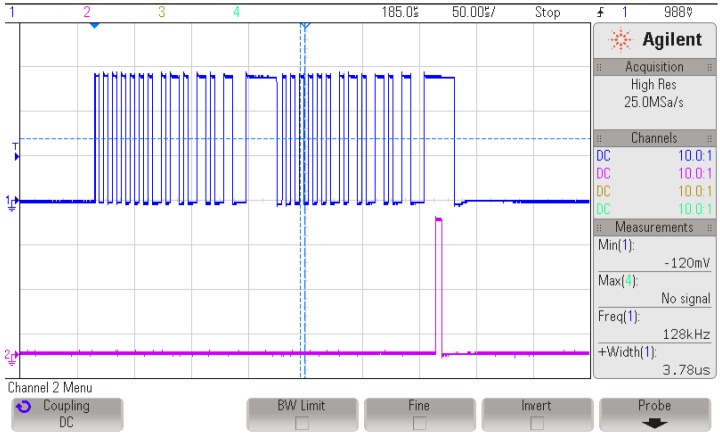
Oscilloscope capture of HCMP output and PIC interrupt during a WuPt decoding.

The PIC12 uses the internal high frequency oscillator (HFINTOSC) and the internal medium frequency oscillator (MFINTOSC). HFINTOSC can be as high as 32 MHz, while MFINTOSC can achieve a maximum of 500 kHz. Configuring the HFINTOSC with 16 MHz allows maximum processing speed at which the MCU demands a power $P_{\mathrm{HF}\_16\mathrm{MHz}}=1.26\;mW$ at $V_{cc}$. The oscillator configuration at 32 MHz is not considered because it requires an active phase locked loop (PLL), which needs more than 2 ms to settle [21] by the time PIC12 exits sleep. The PIC12 switches to MFINTOSC at different times of the decoding process where it consumes $P_{\mathrm{MF}\_500\mathrm{kHz}}=200\;\mu W$ at $V_{cc}$. Both oscillators need a warm-up time $t_{warmup}=5\;\mu s$ to stabilize when waking up from sleep. Switching between MFINTOSC and HFINTOSC and vice versa requires a time slot of $t_{oscsw}=2\;\mu s$. Moreover, the designed LNA’s turn-on time $t_{\mathrm{lnaON}}$ requires less than 1 μs. The BBAMPS RC time constants set the time $t_{\mathrm{bbampsON}}=20\;\mu s$ it needs to settle. Finally, the HCMP powers-on in $t_{\mathrm{hcmpON}}=1\;\mu s$. Upon exiting sleep, the PIC12 uses MFINTOSC as the main oscillator, then it enables the BBAMPS and holds, waiting for $t_{\mathrm{bbampsON}}$. Next, it enables the LNA and HCMP at once then switches to HFINTOSC. By this time, all peripherals are ready to receive the WuPt. The MCU, then, waits for $t_{p}$, then operates as described in Section 3.5. Figure 15 shows an oscilloscope screen capture of a WuPt decoding. The first channel represents the HCMP’s output, while the second is the interrupt generated by the PIC12. It indicates a successful WuPt pattern correlation. For the sake of the WuRx’s evaluation, the different operation parameters are selected, $f_{\mathrm{BB}}=128\;k\mathrm{Hz}$, $t_{\mathrm{SB}}=23\;\mu s$, k=2 for 16-bit pattern, $T_{\mathrm{WF}}=N_{\mathrm{WuPt}}T_{\mathrm{WuPt}}$ and $T_{S}=32\;ms$. Therefore, the total needed power-on time $t_{\mathrm{POWER}}$ of the WuRx is given by:

(25) $$\begin{array}{c} t_{\mathrm{POWER}}=t_{warmup}+t_{\mathrm{bbampsON}}+t_{oscsw} \end{array}$$

From Equations (23) and (24), $T_{\mathrm{ON}}=55\;\mu s$ is chosen. The average power consumption of the WuRx $P_{WURX}$ during $T_{\mathrm{ON}}$ is calculated in the following equation.

(26) $$\begin{array}{c} P_{WURX}=P_{W\mathrm{DT}}+\frac{\tau_{1}P_{\mathrm{MF}\_500\mathrm{kHz}}+\tau_{2}P_{\mathrm{BBAMPS}}+\left(t_{oscsw}+t_{p}\right)P_{x}}{T_{\mathrm{ON}}} \end{array}$$

where:

$$\begin{array}{c} \begin{array}{cc} \tau_{1} & =t_{warmup}+t_{\mathrm{bbampsON}}\;,\;\\ \tau_{2} & =t_{\mathrm{bbampsON}}+t_{oscsw}+t_{p}\;\mathrm{and}\;\\ P_{x} & =P_{\mathrm{LNA}}+P_{\mathrm{HCMP}}+P_{\mathrm{HF}\_16\mathrm{MHz}} \end{array} \end{array}$$

Table 2 summarizes all timing parameters of the sensor node.

**Figure 16:**
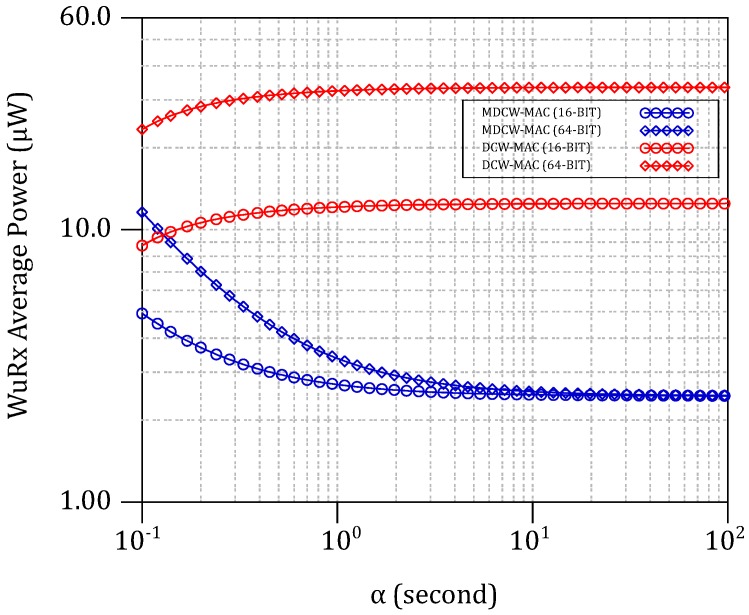
Average WuRx power consumption of the DCW-MAC and MDCW-MAC when decoding 16-bit and 64-bit WuPt.

The power and the timing parameters are either measured or retrieved from every device’s datasheet. The MDCW-MAC energy models proposed in Section 2 are used to calculate the average power consumptions per α interval along with a comparison with DCW-MAC. Figure 16 plots the simulated average power consumption of the NdRx1’s WuRx, $P_{l\_rx}=E_{l\_rx}/\alpha$ for 16-bit and 64-bit WuPts. Using the MDCW-MAC, the WuRx consumes 2.8 μW for α>10 s for both 16-bit and 64-bit WuPts. Because of the reduced channel listening of the WuRx (i.e., $T_{\mathrm{ON}}$), the power consumption is much reduced comparing to the DCW-MAC protocol. In DCW-MAC, the increasing of the WuPt’s ID length leads to an increased average power consumption. Furthermore, assuming a WSN with N nodes, the impact of the WuRx consumption on the entire WSN when using either DCW-MAC or MDCW-MAC is compared.

**Figure 17:**
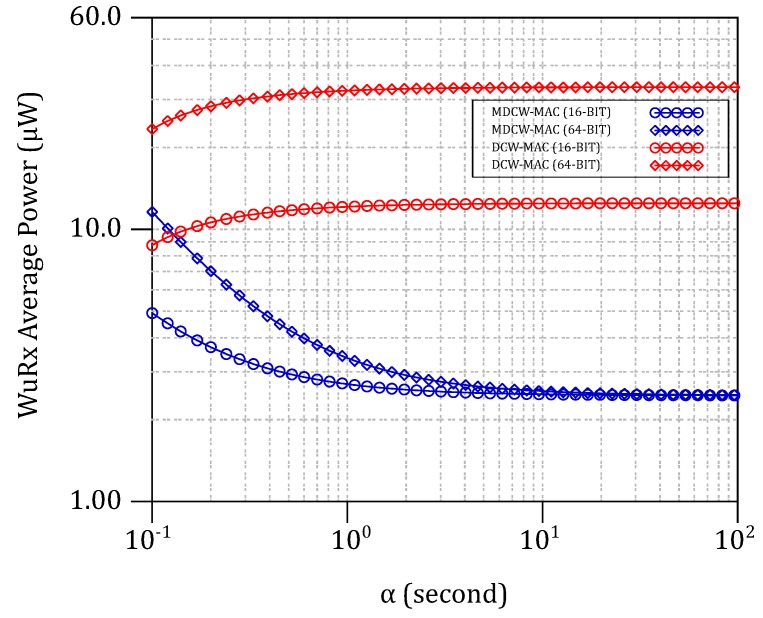
MDCW-MAC and DCW-MAC average power consumptions per node against the α interval for different WSN configurations.

Taking the cases where N=2 and N=1024 then replacing them in Equation (10), Figure 17 plots the simulated mean power consumption P=E/αN of a single node per α. When N=2 and $\alpha \geq 10^{4}\;s$, *P* reaches its lowest value, yielding 8.8 μW and 28.1 μW for MDCW-MAC and DCW-MAC, respectively. Likewise, P=7.38 μW and P=14.03 μW when N=1024 and α≥10 s. It can be observed that the influence of the transmitter’s power consumption dominates less as the number of nodes increases (i.e., NdTx). Then, the *P* converges to the average consumption of the WuRx plus the minimum power required for the sleep state. From the above interpretations, *P* is roughly three-times less with MDCW-MAC than that of DCW-MAC. For low traffic (α↗↗), the network significantly reduces the average energy consumption while taking advantage of WuRx’s listening readiness. The parameters $T_{\mathrm{ON}}$, $T_{S}$ and $T_{d}$ directly affect the above figures, as well as the latency required for WuPt detection. Until now, $T_{d}$ was chosen $2\;T_{\mathrm{WuPt}}$ as mentioned in Table 2.

**Figure 18:**
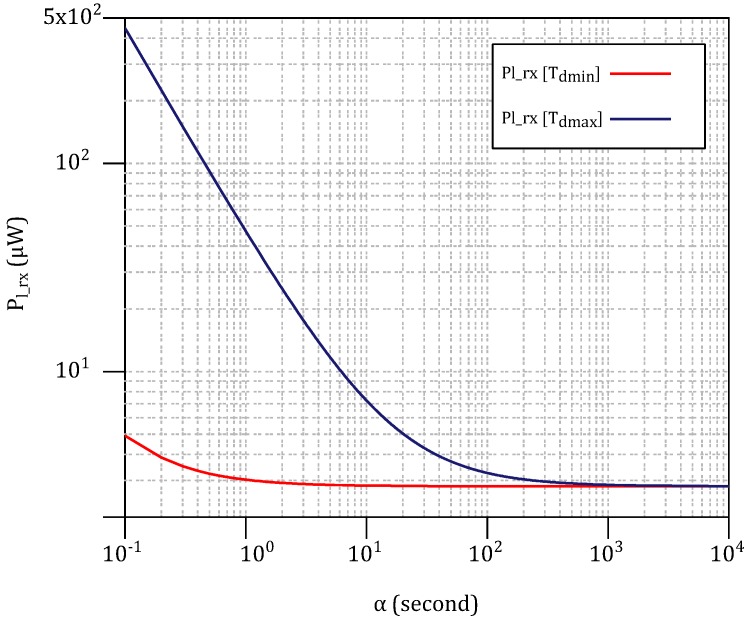
Average power consumption of the Ndrx1’s WuRx ($P_{l\_rx}$) against the variations of α and $T_{d}$.

In a real case with the presence of a WuPt and excessive noise/interferences, the WuRx will continuously try to detect a WuPt until it reaches the end of the WF, if the WuRx manages to detect the preamble, resulting in a longer decoding time. Therefore, $T_{d}$ ultimately changes within the range of $\left[2\;T_{\mathrm{WuPt}},T_{\mathrm{WF}}\right]$. However, $T_{d}$ can still be limited by the DBB if the power consumption is prioritized over the detection convenience. Figure 18 illustrates the expansion of $P_{l\_rx}=E_{l\_rx}/\alpha$ with the maximum and minimum value of $T_{d}$ (i.e., $T_{dmin}$ and $T_{dmax}$), where $T_{dmin}=2\;T_{\mathrm{WuPt}}$ and $T_{dmax}=T_{\mathrm{WF}}$. The difference is significant at low α.

Moreover, the minimum theoretical sensitivity of the WuRx sets the minimal detectable signal. A proper operation requires a higher SNR margin to compensate for the detection imperfections. For instance, the preamble detection process represents a critical step in designing the WuRx. A poor detection mechanism will result in packet errors and degraded noise immunity. Furthermore, the figure of the WuRx’s sensitivity is measured by placing an attenuator between the WuRx and a WuTx, all connected with 50-ohm shielded coaxial cables. The WuTx transmits $N_{\mathrm{WFTX}}$WF with power output of −30 dBm in burst mode. For every successfully decoded WuPt, the PIC12 issues an interrupt to the CC430. $N_{\mathrm{INT}}$ denotes the total number of interrupts. Afterwards, those interrupts are logged and compared to the total number of transmitted WuPts.

A time slot of 10 ms exists between two transmitted WuPts to allow enough time for WuPt processing. The process is repeated for every attenuation step of 2 dB. To have a practical figure of the WuRx’s sensitivity, the packet error rate (PER) is measured in every iteration. The PER can be calculated as follows:

(27) $$\begin{array}{c} \mathrm{PER}=(1-\frac{N_{\mathrm{INT}}}{N_{\mathrm{WFTX}}}) \end{array}$$

**Figure 19:**
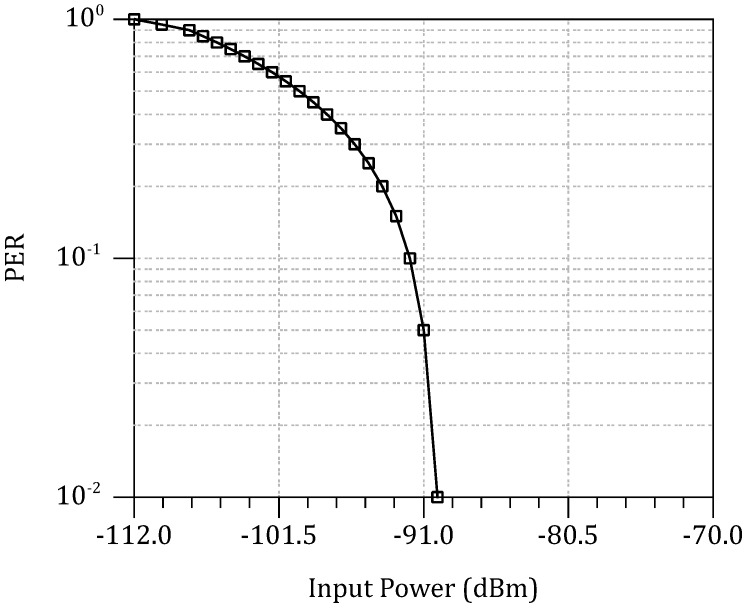
Packet error rate against the WuRx’s input power.

Hence, from Equation (27), the PER can be plotted against the input power of the WuRx as shown in Figure 19. In this design, $\mathrm{PER}=10^{-2}$, which corresponds to −90 dBm, is sufficiently tolerated. Therefore, the sensitivity of the WuRx is considered −90 dBm.

To confirm the obtained results, a line-of-sight range test was performed using both internal antennas with a gain of −1 dBi. With a transmission power of 7 dBm, a successful WuPt is observed at a distance coverage of more than 800 m. Table 3 compares most recent WuRx works. Given that all of them are designed differently, a generic figure of merit cannot compare them fairly. For instance, energy-per-bit analysis expels the sensitivity metric. It becomes irrelevant as it is agreed that high sensitivity and low power consumption for WuRxs are the main concerns for an adequate performance.

## 5. Conclusions

In this work, a MAC protocol and the design of WuRx are presented. The MDCW-MAC is optimized to allow brief channel listening, so as to decrease the average energy consumption of the WuRx. The reduced listening period affects the WSN average energy consumption. The WuRx consists of LNA, SLD, BBAMPS, HCMP and a DBB. The design details of all blocks are discussed separately. A proof-of-concept on PCB was realized to evaluate the WuRx’s operation within a sensor node. The obtained WuRx consumes around 2.8 μW for low to mid-range packet arrival intervals. The LNA contributed in enhancing the WuRx’s sensitivity, reaching −90 dBm. The incorporated digital baseband is based on a low power MCU and offers two functionalities. First, it implements the MDWC-MAC protocol. Secondly, it adds the addressing capability to the WuRx with a scalable data rate and ID length. In terms of energy consumption, the MDCW-MAC outperforms the DCW-MAC because of the reduced listening time. The performed energy analysis of the entire WSN reveals the benefit of adopting the WuRx technology over the conventional radio duty-cycle. 

## Figures and Tables

**Table 1:** Sensor node power parameters. HCMP, hysteresis comparator; LPM3, Low-power Mode 3.

Power	Description	Value (μW)
$P_{\mathrm{LNA}}$	Active LNA	1000
$P_{\mathrm{BBAMPS}}$	Active BBAMPS	26.3
$P_{\mathrm{HCMP}}$	Active HCMP	72
$P_{\mathrm{HF}\_16\mathrm{MHz}}$	Decoder clocked at 16 MHz	1260
$P_{\mathrm{MF}\_500\mathrm{kHz}}$	Decoder clocked at 500 kHz	200
$P_{tx}$	Power needed for data Tx ( 10 dBm)	$66\times 10^{3}$
$P_{rx}$	Power needed for data Rx	$32\times 10^{3}$
$P_{buck}$	Buck converter power consumption	1
$P_{\mathrm{WSleep}}$	WuRx’s lowest sleep state	0.04
$P_{\mathrm{MSleep}}$	CC430 in LPM3	1
$P_{\mathrm{RFSW}}$	RF switching chip	0.2

**Table 2:** Sensor node timing parameters.

Parameter	Description	Value(μs)
$T_{tx}$	Needed time for Data Tx	$25\times 10^{3}$
$T_{rx}$	Needed time for Data Rx	$25\times 10^{3}$
$T_{ack}$	Acknowledgment slot duration	$2\times 10^{3}$
$T_{tx\_rx}$	Switch from Tx to Rx and vice versa	2
$T_{H}$	Delay before sending Ack	<1 $\times 10^{3}$
$T_{\mathrm{SW}}$	Radio turn-on delay	1
$T_{\mathrm{ON}}$	WuRx in preamble scanning	55
$T_{S}$	WuRx in inactive state	$32\times 10^{3}$
$T_{d}$	Minimum WuPt detection duration	$2\;T_{\mathrm{WuPt}}$
$T_{\mathrm{WuPt}}$	Duration of a single WuPt	140
$T_{\mathrm{WF}}$	Duration of multiple WuPt	$32.2\times 10^{3}$

**Table 3:** WuRx prototypes comparison. TRF, tuned-RF; PRFD, passive RF detector, SR, super-regenerative; SH, superheterodyne.

	This Work	[3]	[10]	[7]
Frequency (GHz)	0.868	0.868	0.868	0.868
Listening Power (μW)	2.8 ${}^{\psi }$	1.2	40	3–86.7
Processing Power (μW)	$1.38\times 10^{3}$	63	40	$27.5\times 10^{3}$
Sensitivity (dBm)	−90	−55	$-97{\;}^{†}$	−83
Data Rate (kbps)	128	-	1.25	0.06–8
Architecture	TRF	PRFD	SR	SH
Implementation	OtS *	OtS	OtS	130 nm

* Off-the-shelf; ${}^{\psi }$ $\alpha >10^{1}\;s$, $T_{\mathrm{ON}}=55\;\mu s$ and $T_{S}=32\;ms$; ${}^{†}$ PER = $10^{-3}$.
